# Cardiovascular magnetic resonance findings in repaired anomalous left coronary artery to pulmonary artery connection (ALCAPA)

**DOI:** 10.1186/1532-429X-13-27

**Published:** 2011-05-16

**Authors:** Aurelio Secinaro, Hopewell Ntsinjana, Oliver Tann, Pia K Schuler, Vivek Muthurangu, Marina Hughes, Victor Tsang, Andrew M Taylor

**Affiliations:** 1 Cardiorespiratory Unit, UCL Institute of Cardiovascular Sciences & Great Ormond Street Hospital for Children, London, UK; 2 Department of Imaging, Bambino Gesù Children's Hospital, Rome, Italy

## Abstract

**Background:**

Anomalous origin of the left coronary artery from the pulmonary artery (ALCAPA) is a rare coronary artery anomaly. This study shows the role of cardiovascular magnetic resonance (CMR) in assessing young patients following surgical repair of ALCAPA.

**Methods:**

6 patients, aged 9-21 years, with repaired ALCAPA (2 Tackeuchi method, 4 direct re-implantation) underwent CMR because of clinical suspicion of myocardial ischemia. Imaging used short and long axis cine images (assess ventricular function), late-gadolinium enhancement (LGE) (detect segmental myocardial fibrosis), adenosine stress perfusion (detect reversible ischaemia) and 3D whole-heart imaging (visualize proximal coronary arteries).

**Results:**

The left ventricular (LV) global systolic function was preserved in all patients (mean LV ejection fraction = 62.7% ± 4.23%). The LV volumes were within the normal ranges, (mean indexed LVEDV = 75.4 ± 3.5 ml/m^2^, LVESV = 31.6 ± 9.4 ml/m^2^). In 1 patient, hypokinesia of the anterior segments was visualized. Five patients showed sub-endocardial LGE involving the basal, antero-lateral wall and the anterior papillary muscle. Three patients had areas of reversible ischemia. In these 3, 3D whole-heart MRA showed that the proximal course of the left coronary artery was occluded (confirmed with cardiac catheterisation).

**Conclusions:**

CMR is a good, non-invasive, radiation-free investigation in the post-surgical evaluation of ALCAPA. In referred patients we show that basal, antero-lateral sub-endocardial myocardial fibrosis is a characteristic finding. Furthermore, stress adenosine CMR perfusion, can identify reversible ischemia in this group, and was indicative of left coronary artery occlusion.

## Background

Anomalous origin of the left coronary artery from the pulmonary artery (ALCAPA), also known as Bland-White-Garland syndrome, is a rare congenital cardiovascular defect that occurs in approximately 1 in 300 000 live births [1] or 0.25-0.5% of children with congenital heart disease [2]. The mortality of untreated ALCAPA has been estimated to be approximately 90% in the first year of life [3]. However, 5-10% of cases can survive past infancy into adulthood. These older patients can present with myocardial infarction, left ventricular dysfunction, mitral regurgitation, or silent myocardial ischemia, which can lead to sudden cardiac death.

Symptoms and signs usually occur in early infancy (4^th^-5^th ^month of life) when pulmonary vascular resistance drops and left coronary artery flow is reduced. All blood is now supplied by the right coronary artery and if there are few coronary collateral vessels, this can lead to coronary insufficiency. Patients with better coronary collateralization may present later in life with a 'steal' phenomenon: Blood from the right coronary artery passes to the left system via collaterals, and then directly into the low pressure pulmonary artery (systemic to pulmonary shunt), thus leading to under-perfusion in the left coronary territory [4]. Surgery is the only definitive treatment to restore a dual coronary artery circulation, to correct the shunting and to reverse the 'steal' that causes myocardial ischemia.

Depending on the anatomy, the principal surgical options are direct re-implantation (also known as coronary button transfer technique), Tackeuchi repair (where the pulmonary artery is opened, creating an anterior transverse flap of native pulmonary artery tissue, which creates a baffle to carry the aortic oxygenated blood to the anomalous coronary artery [5]), left coronary artery ligation [6] or coronary artery bypass grafting (CABG). Though a transcatheter closure of ALCAPA has been recently reported as a potentially safe and effective alternative treatment [7], direct re-implantation is considered as the first line surgical option nowadays, as it yields a more anatomical position by connecting the left coronary artery (LCA) to aorta.

Once treated, the most feared complication in patients is recurrence of myocardial ischemia and potential narrowing of the surgical anastomosis. However, assessment of these patients may not be straightforward and the interpretation of image findings may be difficult. In this study, we wanted to demonstrate the CMR appearance in a small group of young patients with repaired ALCAPA.

## Methods

### Patients

In this retrospective study, 6 patients with repaired ALCAPA underwent full CMR assessment between may 2009 and March 2010. They were all referred for assessment because of clinical suspicion of myocardial ischemia (mean age 15.3 ± 4.2 years, range at 9.7 and 21.7; 3 female). 4 patients were originally treated with direct re-implantation of the LCA, 2 patients with Tackeuchi repair (see Table 1), with the original operations performed at several centres.

**Table 1:** Patient characteristics

Sex	Age at scan	Weight (kg)	Type of repair	Age at repair	Indication for MR
F	9 y 8 m	30	Direct reimplantation	4 m	Chest pain
F	16 y 10 m	64	Tackeuchi repair	14 m	Suspected LCA baffle obstruction at echo
M	21 y 8 m	104	Tackeuchi repair	6 m	Supravalvar PS and chest pain
F	17 y 5 m	56	Direct reimplantation	17 y 3 m	Chest pain and ECG changes
M	13 y 5 m	36	Direct reimplantation	2 m	ECG changes
M	12 y 7 m	35	Direct reimplantation	12 y 11 m	Syncope during exercise test

All CMR scans were visualized and post-processed by 2 clinical cardiovascular CMR experts, who were aware of the patient's clinical details, but blinded to any other imaging assessment, in particular the conventional coronary catheter angiography.

The study had Institutional ethics approval and all parents and patients gave informed consent for the use of their images and data.

### CMR

Five patients underwent CMR while awake and one patient had general anaesthesia. This scan was performed as a hybrid procedure together with the coronary catheterization in an X-ray/MR hybrid laboratory (consisting of an x-ray Artis biplane system and a 1.5-T Avanto MR scanner, Siemens Medical Solutions, Erlangen, Germany) [8].

#### Cine CMR

Retrospectively gated, balanced steady-state free-precession cine images were acquired in the vertical long-axis and 4-chamber views. They were used to plan the short-axis stack, which included the extent of both ventricles (9 to 12 slices). The cine balanced steady-state free-precession sequence parameters were as follows: TR 2.4 ms, echo time 1.2 ms, flip angle 68°, slice thickness 6-10 mm, matrix 200 × 240, and field of view, 280 to 380 mm, with 25 phases per cardiac cycle. Post-processing assessment of RV and left ventricular (LV) volumes was performed by manually defining the endocardial outline at end diastole and end systole in each of the short-axis cine images (OsiriX version 3.8). The end-diastolic volume (EDV) and end-systolic volume (ESV) were calculated with Simpson's rule for each ventricle, and from these volumes, the stroke volume (SV) and ejection fraction (EF) were derived.

Flow assessment using phase contrast MR sequences was also performed in both the ascending aorta above the aortic valve and the pulmonary trunk above the pulmonary valve. We calculated the degree of mitral valve regurgitation as the LV SV minus the total aortic flow during systole.

#### Stress perfusion imaging

Myocardial perfusion was performed under pharmacological stress with intravenous adenosine in a dose of 140 μg/kg/min over 6 minutes with constant ECG and blood pressure monitoring. The target of cardiovascular stress was either a 20% increase in the heart rate or a symptomatic response (chest tightness, hot flushes, throat pain, breathlessness). The first-pass myocardial signal increase was evaluated during a bolus injection of gadolinium (Dotarem^®^, gadoterate meglumine, Gd-DOTA, Guerbet, Paris, France) at 0.1 mmol/kg, injected in the ante-cubital vein at flow rate of 2-3 ml/s and followed by a saline flush of 25 ml, flow 2-3 ml/s. Short axis images were acquired with a saturation-recovery, spoiled gradient echo sequence in at least 3 slices per heart beat (slice thickness 8 mm) with the following parameters: TI 200 ms, TR 260 ms, TE 1.1 ms, flip angle 12°, matrix 192 × 144, rectangular field of view of 187 × 250 mm, number of excitations 1, acquisition every RR interval. Data from 50 cardiac cycles were acquired. Patients were asked to hold their breath for as long as possible, then revert to shallow respiration during this acquisition, or, for the child requiring general anaesthesia, data was acquired during suspended mechanical ventilation. The perfusion sequence was repeated after 15 minutes at rest with the same parameters and bolus injection of gadolinium.

#### Late gadolinium enhanced CMR

Inversion-recovery late gadolinium enhanced (LGE) CMR was performed after the rest perfusion intravenous injection of gadolinium (0.1 mmol per kilogram of body weight) by using a two-dimensional T1-weighted turbo field-echo technique in the cardiac short and long-axis planes (TR 2.9, TE 1.3, flip angle of 50°, slice thickness of 8-10 mm, matrix 256 × 192, field of view of 350 mm). The inversion time was adjusted for optimal suppression of signal from normal myocardium, and the images were obtained within 10-15 minutes after injection (inversion time approximately 250-350 ms). All LGE images were interpreted accordingly to the American Heart Association 17-segment model [9].

#### 3D whole-heart MRA

Coronary artery imaging was achieved using a 3D whole-heart MRA sequence covering the entire heart were obtained in a sagittal orientation by using a magnetization-prepared, 3D balanced, steady-state free precession sequence (TR 3.0, TE 1.5, flip angle 90°, number of lines per segment acquired per cardiac cycle 30-40, sensitivity-encoding factor 2.0, bandwidth per pixel 590 Hz, field of view 280 × 280 × 120 mm, acquisition matrix 192 × 192 × 80 and iso-volumetric voxel size 1.5 × 1.5 × 1.5 mm). Magnetization preparation was achieved by applying a T2-weighted preparation pulse with an echo time of 50 ms and a frequency-selective fat-saturation pulse followed by a spoiler gradient. A customized shim procedure was applied to the volume of the entire heart. For real-time respiratory gating and real-time correction of the 3D volume in the craniocaudal direction, a navigator echo was acquired from a cylindrical region that was generated by a two-dimensional excitation pulse perpendicular to the right hemi-diaphragm with a gating window of ±3 mm. The navigator efficiency (%) and total scan time (minutes) was calculated for each patient.

## Results (see Table 2)

**Table 2:** MR findings and results.

LV EF (%)	**LV EDVi (ml/m**^**2**^**)**	Regional WMA	LGE	Distribution of LGE	MR	MV prolapse	PS	Inducible ischemia	MRCA	X-ray coronary angiography
60	76	No	Yes	Sub-endocardial circumferential & PMs	Trivial	Yes	No	Yes	Occluded LCA	Occluded LCA
61	69	No	No	-	No	Yes	Mild	No	Patent LCA	Patent LCA
60	79	No	Yes	Sub-endocardial antero-lateral & PMs	Trivial	no	Moderate	No	Patent LCA	Not performed
62	78	Yes	Yes	Sub-endocardial anterior & PMs	No	Yes	No	Yes	Occluded LCA	Occluded LCA
61	101	No	Yes	Sub-endocardial anterior & PMs	Mild	No	No	No	Non diagnostic	Not performed
72	75	No	Yes	Sub-endocardial anterior & APM	Mild	Yes	No	Yes	Occluded LCA	Occluded LCA

LV EF - left ventricular ejection fraction (%), LV EDVi - indexed left ventricular end-diastolic volume (ml/m^2^), WMA - wall motion abnormality, LGE - late gadolinium enhancement, PM - papillary muscles, APM - anterior papillary muscle, MR - mitral regurgitation, MV - mitral valve, PS - pulmonary stenosis, LCA - left coronary artery.

### Cardiac function at rest

The LV ejection fraction (EF) was preserved in all patients (62.7 ± 4.23%). Cardiac volumes were within the normal ranges (mean body surface area-indexed LV end-diastolic volume = 79.6 ± 10.0 ml/m^2^, mean LV end-systolic volume = 33.0 ± 9.1 ml/m^2^). Only one patient had mild dilatation of the LV in the presence of combined moderate aortic valve regurgitation and mild mitral valve regurgitation. One patient had isolated, mild hypokinesis of the mid-ventricular anterior segment, otherwise normal LV wall motion at rest was found in all cases.

There was a trivial to mild degree of mitral regurgitation in 4 of the 6 patients. Four patients had bi-leaflet mitral billowing/prolapse. Two of these patients did not have discernable mitral regurgitation either on cine images or from combined volume and flow quantification. The two cases operated with the Takeuchi repair had mild and moderate degrees of pulmonary supravalvar stenosis, respectively.

### Myocardial tissue characterization

**Figure 1:**
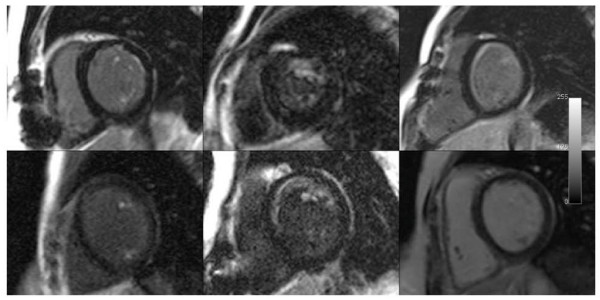
**Late gadolinium enhancement (LGE) images from all 6 patients**. The bottom right image shows normal myocardium. The left and middle columns show enhancement in both papillary muscles, with enhancement in the anterior papillary muscle. There are varying degrees of anterior sub-endocardial LGE in the 5 abnormal images.

There was a consistent LGE pattern present in 5 of the 6 patients (Figure 1). This fibrosis characteristically involved a sub-endocardial layer of the basal, anterior (segment 1) and antero-lateral (segment 6) segments, including both papillary muscles. There was some extension into the septum (segment 2) and into the mid portion of the ventricle (segments 7 & 12) in individual cases.

### Myocardial stress perfusion

**Figure 2:**
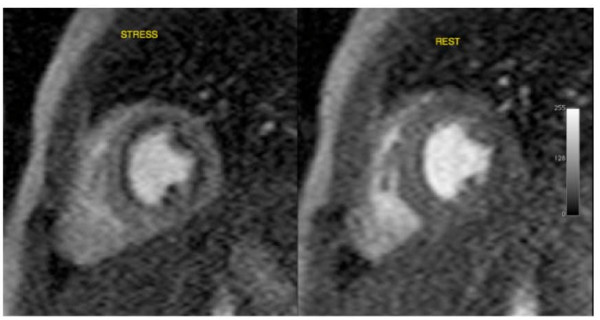
**Adenosine MR stress perfusion imaging shows reversible sub-endocardial ischaemia of the anterior, septal and lateral walls and the papillary muscles at the base of the heart - stress and rest images shown**.

**Figure 3:**
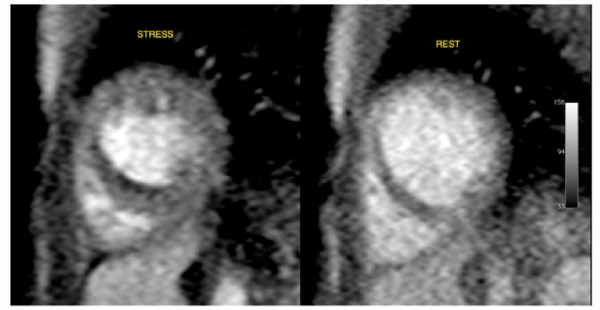
**Adenosine MR stress perfusion imaging shows reversible sub-endocardial ischaemia of the mid-septum at the base of the heart - stress and rest images shown**.

In 3 patients there was myocardial ischemia. This was extensive in all 3 patients; with 50% sub-endocardial ischemia of the antero-septal and antero-lateral segments (Figure 2). These hypo-perfused areas during stress were larger than the area of discrete fibrosis seen on LGE imaging. In one of these patients, there was a further small perfusion defect of the sub-endocardium of the mid-LV, mid-septum. This circumscribed finding was strongly suggestive of ischemia (Figure 3).

### Coronary 3D imaging

In 5 cases, we obtained good quality 3D whole-heart MRA. The mean navigator echo scan efficiency for all patients was 39% [range 27 to 54%], with a mean total scan time of 9 mins 42 s [range 5 mins 55 s to 14 mins 47 s].

**Figure 4:**
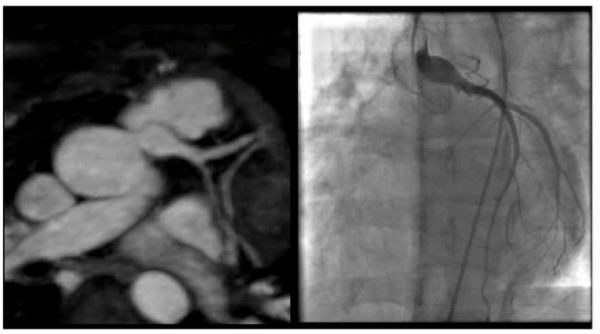
**3D b-SSFP whole heart images, magnetic resonance coronary angiography MRCA (left) shows large patent, reconstructed, re-implanted left coronary artery into the left coronary sinus**. Findings confirmed on X-ray coronary angiography (right).

For the 2 patients who had previously undergone Takeuchi repair, 3D whole-heart MRA imaging demonstrated unobstructed trans-pulmonary coronary baffles. One of these patients had X-ray coronary angiography confirmation of this baffle patency (Figure 4). Neither of these patients had reversible perfusion defects.

**Figure 5:**
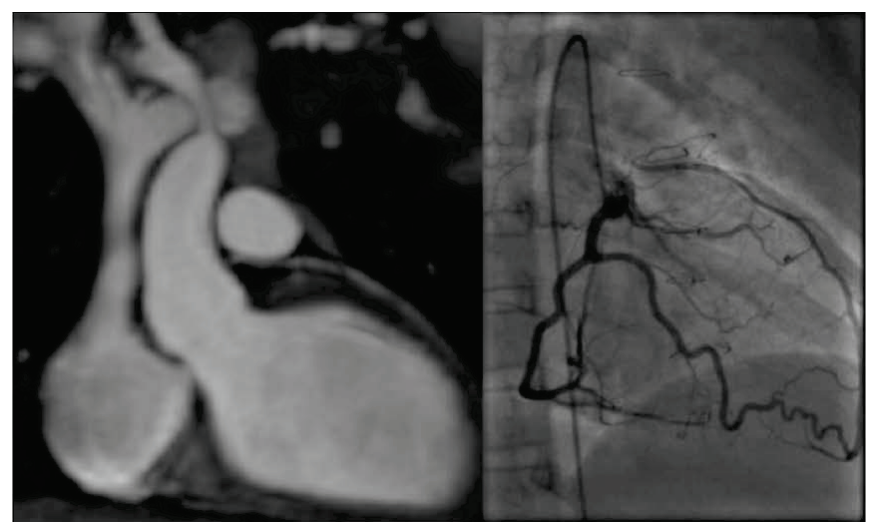
**3D b-SSFP whole heart images, MRCA (left) shows no direct connection between the left coronary artery and the left aortic sinus of Valsalva**. Findings confirmed on X-ray coronary angiography (right).

For the 3 other patients with direct re-implantation, multi-planar reformatted views suggested obstruction of the re-implanted LCA on the MRCA. As these patients had significant adenosine perfusion defects, conventional X-ray coronary angiography was subsequently performed. X-ray coronary angiography confirmed obstruction at the surgical anastomosis in 2 cases and significant stenosis in the other case (Figure 5).

In the final patient, the 3D whole-heart MRA images were technically sub-optimal and the left coronary system was not visualised. However, as this patient minimal clinical symptoms and had no reversible defect on adenosine stress perfusion imaging, X-ray coronary angiography was not performed.

## Discussion

In this study we describe the use CMR to ascertain the morphological and functional status of young patients with suspected on-going myocardial ischemia after surgical repair of ALCAPA.

We show that at rest, global left ventricular systolic function is normal, with only one patient showing mild left ventricular segmental hypokinesia. Furthermore, we show that these patients have a characteristic sub-endocardial LGE pattern, with fibrosis of the papillary muscles and basal antero-lateral myocardium. This pattern was seen in 5 of 6 patients and would be consistent with the brightly echogenic myocardium seen during echocardiography in neonates with this condition [10]. Furthermore, the pattern of LGE we observed is in keeping with animal observation [11], but has a different localization to that observed by Browne and colleagues in 2 neonates referred for cardiac transplantation - predominantly apical LGE [12].

We also show that a combination of CMR coronary imaging and adenosine-stress perfusion imaging [13], can identify patients with significant left coronary artery disease, in keeping with the use of stress perfusion imaging in adult coronary artery disease [14]. Importantly, it is the origin and proximal course of the coronaries that are important to visualise. The morphology of this proximal region of the coronary arteries has previously been shown to be well visualised by MRCA in groups of patients with both anomalous coronary artery disease [15-17] and other types of congenital heart disease [18,19].

The pattern of reversible myocardial ischemia in these patients was sub-endocardial and adjacent to the areas of LGE. For the 5 patients in whom the coronary arteries were clearly seen at CMR, the presence of a significant perfusion defect was associated with coronary artery compromise.

Finally, it should be noted that a reversible perfusion defect could potentially occur in the presence of normal coronary morphology (4) on 3D whole-heart MRA. Conceivably, such a pattern of ischemia may be a consequence of the collateral circulation that developed prior to repair. This may not represent the current luminal state of the coronary arteries, and may not require treatment. Alternatively, others have suggested that these areas of perfusion defect may represent areas of microvascular disease [20,21].

## Limitations

This study is limited as it is retrospective and descriptive of an inhomogeneous patient population (in terms of age and previous operative type). However, the CMR findings are consistent across this small patient group, who have all been exposed to a similar pathophysiological insult.

## Conclusions

CMR can provide a comprehensive assessment of the anatomical, functional and ischemic consequences of coronary artery disease in young patients following surgical repair of ALCAPA.

The finding of a basal, antero-lateral late-gadolinium enhancement pattern, including the papillary muscles, should prompt consideration of this diagnosis in paediatric or adult patients with this finding, following a presentation with chest pain.

In this small study, adenosine perfusion MR can detect occluded ALCAPA re-implantation, though ultimately, larger and longitudinal studies of this condition are required to establish the true prognostic importance of imaging findings. Due to the low prevalence of ALCAPA, this will require multicentre participation.

## Competing interests

The authors declare that they have no competing interests.

## Authors' contributions

AS, HN, OT, PKS, VM and MH acquired the MR data. VM optimised the MR sequences. AS, VT and AMT participated in the design and coordination of the study. AS and AMT drafted the manuscript. All authors read and approved the final manuscript.
